# Doom or Deliciousness: Challenges and Opportunities for Visualization in the Age of Generative Models

**DOI:** 10.1111/cgf.14841

**Published:** 2023-06-27

**Authors:** V. Schetinger, S. Di Bartolomeo, M. El‐Assady, A. McNutt, M. Miller, J. P. A. Passos, J. L. Adams

**Affiliations:** ^1^ TU Wien; ^2^ Northeastern University; ^3^ ETH AI Center; ^4^ University of Chicago; ^5^ University of Konstanz; ^6^ Hugging Face

## Abstract

Generative text‐to‐image models (as exemplified by DALL‐E, MidJourney, and Stable Diffusion) have recently made enormous technological leaps, demonstrating impressive results in many graphical domains—from logo design to digital painting to photographic composition. However, the quality of these results has led to existential crises in some fields of art, leading to questions about the role of human agency in the production of meaning in a graphical context. Such issues are central to visualization, and while these generative models have yet to be widely applied in visualization, it seems only a matter of time until their integration is manifest. Seeking to circumvent similar ponderous dilemmas, we attempt to understand the roles that generative models might play across visualization. We do so by constructing a framework that characterizes what these technologies offer at various stages of the visualization workflow, augmented and analyzed through semi‐structured interviews with 21 experts from related domains. Through this work, we map the space of opportunities and risks that might arise in this intersection, identifying doomsday prophecies and delicious low‐hanging fruits that are ripe for research.

## 1. Introduction

Generative models are increasingly prominent in many domains. There has been astonishing technological development among such tools, such as the text‐to‐image generation of DALL‐E 2 [Ope22a], Stable Diffusion [RBL^*^22], or MidJourney [Mot22], as well as the textual synthesis found in tools like GPT‐3 [Ope22b] or Copilot [Git22]. These systems transform a (typically textual) prompt and into an entity (such as an image or text) drawn from their learned representations of training data. These tools have been hailed for their rapid development and high‐quality results.

**Figure:**
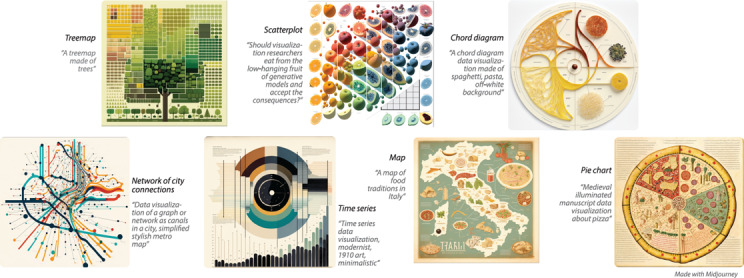
Figure 1: Different types of data visualization (and the prompts used to create them) as imagined by text‐to‐image generative models. While these examples are delicious (in their graphical intrigue and exploration of style), they risk doom by potentially creating misplaced trust.

Yet, this evolution has not been without friction. The automated creation of images and text raises questions about the role of human agency in the production of meaning in a graphical context, creating tensions in professional [Plu22], legal [Vin22], and artistic [Roo22] contexts. For instance, the term *Bach Faucet* [Com22] captures the paradox that the existence of a machine that can produce arbitrarily many high‐quality instances of an artistic style reduces the value of that artistic style—inspired by an early success in generative art that was able to produce sonatas in the style of Bach [Cop04]. Like other models, generative models replicate biases present in their training data, which might yield racist imagery [Ope22c].

Despite these challenges, these models have the potential to improve a wide variety of visualization workflows, such as by increasing the speed of production, facilitating creativity, and enabling expression. However, there has been little investigation into the role these generative models might play in visualization. Wood [Woo22] called for their use as a way to break away from the “*walled garden*” of structured visualization, while others have explored using the visualization generation as means to explore the possibility space of highly stylized visualizations [For22, SFPM^*^22] or validating visualization usage [WTC22]. While some are skeptical [Ma23] about the holistic utility of such models, we believe that their wider spread utilization is on the nearby horizon.

This work seeks to circumvent the dilemmas found in other domains' interaction with generative models by trying to understand this landscape before it emerges. In support of this goal, we seek to answer:

*What challenges and opportunities might we expect to find as use of generative models becomes commonplace in visual design and analytical workflows?*

We answer this question by seeking the concerns, opinions, and predictions of domain experts (*N* =21) from visualization, machine‐learning, art, and art history in a semi‐structured interview study (Sec. 3). Through this study, we elicited beliefs about the risks and opportunities posed by the generative models. Our interview study principally focuses on image‐based generative models (IGMs); however, there are a wide variety of other generative models, such as text‐based ones (e.g. chatGPT [Ope23]).

**Figure 2:**
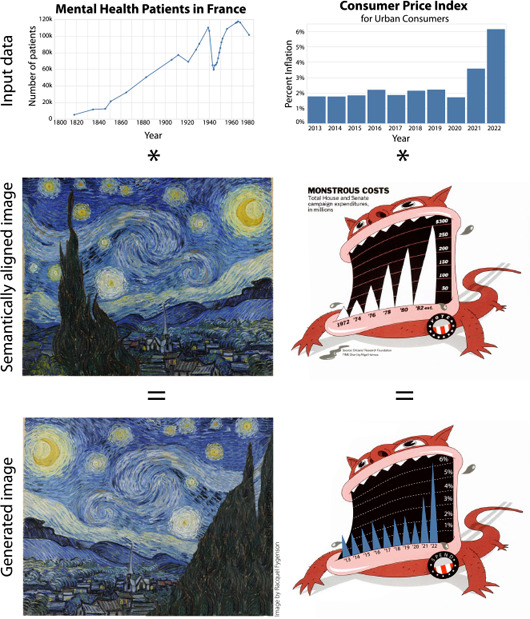
Future IGMs will likely be able to incorporate data or charts as part of their generation process. Racquel Fygenson conceptualized using an IGM to blend data with a thematically relevant image (left). IGMs could also be used to restyle well‐known visualizations (right). Data from [CC10] (left) and [oLS23] (right).

We analyze these findings by locating challenges and opportunities available at each stage of a standard visualization pipeline (Sec. 4). We find that participants believed that the use of generativity in visualization was promising. For instance, it may allow us to capture ephemeral aspects of visualization (such as emotion), or to create artistically rendered visualizations (as in Fig. 2), or to increase the speed with which visualization designers can rapidly prototype their scientific and graphical communications. Such models also offer ample risk for visualization. For instance, there was substantial concern over the proclivity of such tools to amplify bias and to parrot components of their training data (in such a manner that was not respectful of copyright). We extend these concerns and highlight how future work might ameliorate such issues. In additon, our supplemental material is available on osf.io.

In conducting this study, we seek to provide a forward‐looking foundation of how these models might be used—both to guide future tool construction, but also to steer design of the models themselves. Per Bender and Koller [BK20], this work aims at highlighting the right hills to climb and valleys to watch out for.

## 2. Background and Related Work

From the creation of Generative Adversarial Networks (GANs) in 2014 [MO14] to new text‐to‐image tools in 2022, such as DALL‐E 2 [RDN^*^22], Imagen [SCS^*^22], Stable Diffusion 2.0 [RBL^*^22], and MidJourney [Mot22], the development of image‐based generative models (IGMs) has been progressing at breakneck speed, quickly entering many domains. We posit that it is reasonable, too, to expect the widespread adoption of generative technologies in visualization creation within the coming decade. Similar studies as our own have been carried out in other domains, including newsrooms graphics [LQC22], visual marketing [MV22], computational notebooks [MWDD23] and construction scheduling [PMdS23]. Our study differs from these its focus on the particular visualization practice and design.

Our work is situated among prior studies on generative models, particularly those focused on using generative models to enhance the overall process of visualization (“Gen4Vis”).

The use of generative tools in the development of data visualizations has been growing. Often, in traditional visualization pipelines, a designer has to either be proficient in a programming language, or translate their ideas into tool‐specific operations [SSL^*^22], which makes for a steep learning curve. A number of tools seek to simplify the process, using either visual interfaces [MC21], or, more recently, natural language [SLJL10,MS23,DBSSD23,WCA23] interfaces, which allow users to produce visualizations by simply typing or speaking their questions or requests. Recent surveys [WCWQ22,WWS^*^22, WH22] have explored how machine learning is being applied to the data visualization process. Our work builds on these by looking forward and focusing on generative tasks. Wang et al. focused on dividing applications of ML for visualization into data, visualization, and user [WCWQ22]. The survey presents these elements as modular components of a prospective visualization process pipeline, which has informed our thinking in Sec. 4. Data transformations such as dimensionality reduction could be applied prior to visualization [WFC^*^18]. Machine learning might be applied to a dataset to automatically generate Vega‐Lite visualization specifications [DD18], to highlight issues with those specifications [WTC22], or to suggest the automatic stylistic transfer of graph drawing from one example layout to another [WJW^*^20]. User‐centered interventions might include user profiling, such as predicting a user's next click [OGW19] or anticipating their visual attention across an infographic [BKO^*^17,WCWQ22].

**Table 1:** The predefined potential usages (above the fold) and concerns (below the fold) that participants considered in out study. Items were derived by reviewing common concerns about generative models and by reflecting on our own experience these tools.

Prompt	Description	Question
Design prettyfication	Using a generative model to improve the appearance of a visualization, as in style transfer [SLC^*^22].	Fig. 4, 5A
Embellishment	Automatically introduce visual elements not bound to data. E.g. legends, annotations, decorations, or chart junk.	Fig. 4, 5A
Chart recommendation	Generate charts based on the semantics, types, or shape of the data.	Fig. 4, 5A
Rapid prototyping	Iteratively generate designs to explore different possibilities.	Fig. 4, 5A
Moodboards	An automatically generated visual collage of options for exploring a possibility space.	Fig. 4, 5A
Visualize training data	Aid the creation of generative models by curating content in training data and controlling parameters.	Fig. 4, 5A
Amplifying biases	IGMs, like all ML‐models, are susceptible to bias based on their training data. For instance, a prompt asking for a doctor might only produce images of white men [Ope22c].	Fig. 5B
Untrustworthy results	Charts generated by probabilistic models may not accurately represent the input data or may manipulate it in subtle or confusing ways	Fig. 5B
Replicated private data	Models are well known to parrot [BGMMS21] their training data, and these models run a similar risk.	Fig. 5B
Rip off existing vis style	Just as IGMs are criticized for replicating artists' styles [Hei22], these might try to parrot well known graphics.	Fig. 5B
Replacing vis designers	Sufficiently advanced models might be able to replace human visualization designers	Fig. 5B

The dual of using generativity for visualization (“Vis4Gen”) has also been well studied. These prior works cover topics such as how surrogate models can be used to enhanced interpretbility [MQB19], how visualization can make high‐dimensional structures like embeddings more comprehensible [STN^*^16], and how exposing the internals of black box models can make them easier to understand [HPRC20]. This dichotomy between these approaches informs an axis of our interview study (Fig. 4)—“Gen supports VIS” vs “VIS supports Gen”.

## 3. Methodology: Semi‐Structured Interview Study

To better understand the challenges and risks that may arise through the integration of generative models into visualization workflows, we conducted a semi‐structured interview study that sought to elicit forecasts, opinions, fears, hopes, and concerns arising from experts from various domains. Given the rapid development cycles of generative models, our goal in this study is to prospect the space around visualization—rather than specifically identify the next trends. Our study is informed by the belief that this goal would be best aided by those with expertise in this or related domains.

***Study Participants.*** We conducted interviews with 21 participants, which lasted an average of one hour. Interviews were conducted remotely over Zoom. Participants were drawn from a convenience sample assembled based on their work in relevant domains. In particular, we consulted experts with backgrounds in art or art history (*N* =5), machine learning (*N* =2), and visualization or HCI (*N* =14). We denote each of these backgrounds in participant identifiers as **PX**_*art*_, **PY**_*ML*_, and **PZ**_*vis*_ respectively. Given their central relevance to the topic, most participants' primary background was in visualization, however, most lacked experience with generative tools, and so we supplemented their opinions with those of experts drawn from other fields. We included a participant if they had sufficient expertise in their relevant domain—as demonstrated by holding or pursuing a post‐graduate degree or a substantial history of working in their field. We conducted two pilot interviews prior to the study. Fortunately, the results were sufficiently similar to the other interviews to allow inclusion in our analysis. See appendix for details of self‐reported backgrounds. Additional demographics, such as regarding experience with generative models or with visualization, were not formally collected. Participants were not compensated.

**Figure 3:**
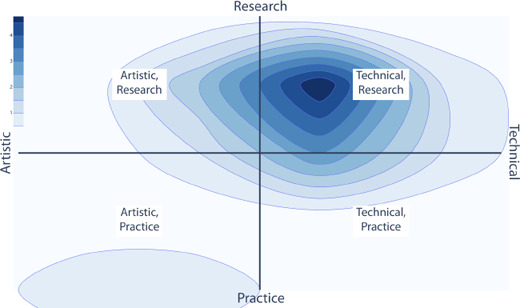
Aggregate participant ratings for self identification.

**Figure 4:**
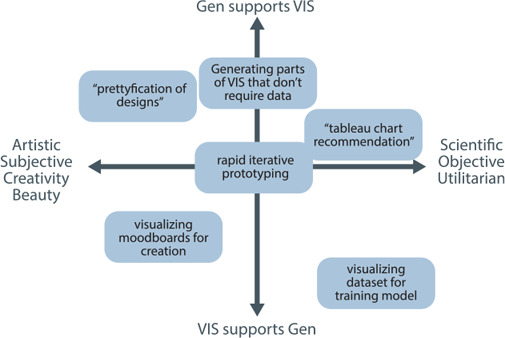
Participants were invited to locate potential usages of IGMs for visualization in a space that sought to elicit how much a concept (such as “rapid iterative prototyping”) was artistic or supported visualization vs. supported generativity (per Sec. 2).

**Figure 5:**
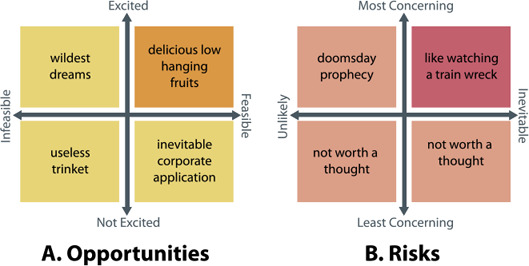
Participants were invited to place both predefined potential usages and concerns for IGMs in visualization (Tab. 1) and self‐defined suggestions onto these axes as a means of ideation. Each quadrant was given a provocative name based on its meaning to help the understanding of the task.

***Study Procedure.*** Interviews consisted of three phases, which, sequentially, sought to elicit (i) participant self‐identification (Fig. 3), (ii) opportunities that they foresaw, and (iii) risks posed. The discussion was focused through a shared Miro board (see appendix for examples), in which participants were invited to create post‐its about ideas and place them on several axes denoting different views (Fig. 4, 5). These predefined prompts (which we list in Tab. 1) were meant to help users ideate on these topics rather than being a comprehensive list of all possible applications or issues. In addition to prompts, participants were invited to create their own and thereby ruminate on their concerns and aspirations for these tools. These discussions were interleaved with situating prompts that reviewed various functionalities possessed by current tools (such as in‐painting [YQS20] and out‐painting [XLC20]) to help situate their thinking among current technologies. We utilized this shared creative space to invite thinking aloud and ideation, which may have been more limited in a more restricted environment. Participants brought up a variety of additional topics (such as semantic chart recommendation), which we discuss in the next section. A complete reproduction of each participant response is available in the appendix.

Through these discussions, participants rated various potential concerns and usages of IGMs on either four axes (purpose, objectivity, excitement, and feasibility) or two axes (concern and likelihood), depending of if we were discussing a potential or a usage, respectively. We focused on these axes (and therein aspects) and set of initial topics (Tab. 1) because they might elicit thoughtful discussion of fears and hopes for these technologies, rather than being an assertion about the relationship between such concepts more generally.

**Figure 6:**
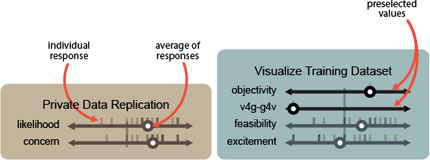
Participant ratings for a potential usage (visualizing training data as part of model construction) and a concern (private data replication). Ticks indicate individual responses, while circles indicate averages. Throughout this paper blue boxes show potential usages, while the brown boxes show concerns.

***Result Coding.*** Interviews were automatically transcribed, and then were coded by two of the authors. Results and themes were then iteratively discussed among the rest of the team in order to form our discussion, which we present in the next section. Given the speculative nature of our work, we sought to locate our findings around a generalizable model, and so we modified a standard visualization pipeline in support of the resultant themes. We augmented this analysis by quantizing the placement of each usage or concern and normalizing it on a unary axis, as shown in Fig. 6. The full results can be found in the appendix, with aggregates presented in Fig. 9.

## 4. Analysis: Help and Harm in the Visualization Pipeline

**Figure 7:**
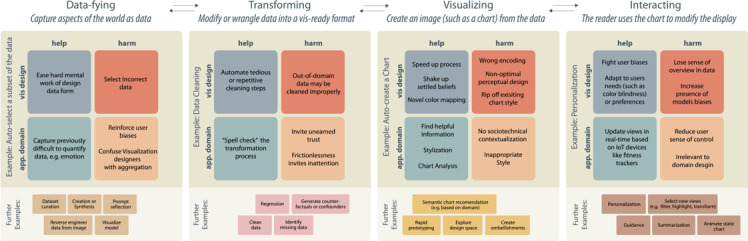
There are opportunities to apply generative models throughout the visualization pipeline. Here, we highlight some of the help and harm that use of generative models can do for particular example tasks at each stage of our broadly organized pipeline.

We now analyze our interviews to identify challenges and opportunities. Per our analysis methodology, described in the previous section, we locate our results within an adapted standard visualization pipeline model [MKC20,Chi00], which we show in Fig. 7. This pipeline consists of four transformations: condensing the world into data by ***Data‐fying*** it (Sec. 4.1), ***Transforming*** (Sec. 4.2) that data into something usable, changing that processed data into an image by ***Visualizing*** (Sec. 4.3), and then using or ***Interacting*** (Sec. 4.4) with that rendered image. These stages are drawn from McNutt et al.'s [MKC20] mirage pipeline, which we refer to for specific definition. While other workflow organizations (such as the KDD model [FPSS96]) might be used, we select this one both to be generalizable but also to be in dialog with other works on applying AI‐tools to visualization [WWS^*^22]. Generative models entail opportunities for useful intervention at each stage of this pipeline.

We further organize our observations following a modification of the SWOT‐strategic management [BEM^*^21], which enumerates the **S**trengths and **W**eaknesses arising from inside an organization, as well as the **O**pportunities and **T**hreats from outside. This approach is often used as part of the decision‐making process for businesses or other organizations. We adapt this analysis by partitioning by whether the opportunities and risks arise from the visualization design side (producer) or the application domain side (consumer). In doing so, we seek to highlight the speculative decision of whether to integrate or implement a particular feature and thereby explore the possibilities available at each stage.

We next traverse our pipeline and highlight the potential that generative models hold for each stage, using the above color scheme to denote risks and opportunities. To support these discussions, we show participant ratings of various potential usages and concerns, such as in Fig. 6.

The majority of this discussion focuses on information visualization‐focused tasks. In addition to the graphical basis of the medium, these results were driven by the cultural prominence of IGMs when we conducted the study. Had we conducted the study several months later, participants may have focused on the capabilities of chatbots like (ChatGPT [Ope23] or Bard [Goo23]) rather than those of IGMs (like DALL‐E 2 [Ope22a]). In addition, our participant pool was mainly drawn from those without experience in domains like Scientific Visualization or animation, which caused our results to be skewed away from such topics. Finally, these considerations over‐emphasize the visualization component of this pipeline (likely motivated by our context as trying to identify opportunities for visualization). However, we note that the other stages also offer ample opportunities for generative intervention.

### 4.1. Data‐fying

Data is not a natural resource, and so its entry into a visualization workflow begins by conceptualizing what that data can be [MKC20]. This process can be time‐consuming and difficult, as data modeling is notoriously challenging [RS19]. A generative model might aid this process by helping the user identify what counts as data (or just by defining the data workspace subsequent analysis will occur in), such as by preparing a SQL query, suggesting a data model, finding a relevant data set, or otherwise injecting its own knowledge [HHS^*^22]. For instance, a natural language prompt such as “get data from earnings of the fourth quarter relative to our competitors” offers ample ambiguity and opportunity for the model to exert agency, such as in identifying who the competitors are, how to compute earnings data, which business units to include, and so on. Huang et al. employ this approach for flow visualization using NLP [HXHT22]. These moments of agency invite risk (e.g., the model might provide bad results or exhibit biases) [GSS^*^22], but they also invite opportunity.

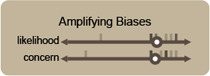

Consider **creating a subset of data** (Fig. 7 left). In such a task, the user asks the model to create a working subset of the data, such as by generating an SQL query for a large database. Such automation might reduce the burden of designing a potentially difficult query and thereby speed up the analysis or design process. In addition, this might also allow for the system to synthesize or create data not present in the original dataset, but that might be inferred from context. These include quantities such as emotions or other ephemeral aspects of the dataset [Woo22]. Such an exchange of agency carries with it potential for harm; for instance, it invisibly surfaces the models' biases. If an idea is out of the scope of the training data, or if a type of person is biased against, that bias will be embedded into the data, possibly without labeling—an error that can cascade down the pipeline, affecting every subsequent data interaction. Reciprocally, the use of a model to construct data might also manifest the biases of the user, such as through confirmation bias. Participants believed that amplifying biases was an inevitable consequence of using these models. For instance, **P21**_*ML*_ noted that *“when you model data using a normal distribution, and then you sample from this normal distribution, you will always have a bias because things that sit closer to the average will have a higher probability of being selected”.*

Similarly, there were concerns about the model parroting its training dataset (Fig. 6 right). Some of these stemmed from anxieties about using the material in ways not permitted by the licenses of the data (an issue which is being examined in the American court system [Vin22]), and the inability to track down the sources used for the generation. For instance, **P6**_*vis*_ observed that *“If you are doing a chart, and you want a simple icon graphic to represent certain subjects, that would be the most common use case, but I have my worries about copyright that comes along with it. If I generate an icon, I don't know if there are any ramifications of me using it in my paper, and that might get me in trouble. So I would be uncomfortable. … I'd be worried that I'm ripping somebody off.”* Such fears are difficult to address in a text‐based medium, but it would be exceptionally quarrelsome to try to check if a particular image has been derived from another, even when using modern reverse image look‐up tools. The recent tool *Have I been trained?* [Spa22] tries to address the issue by offering a lookup function into the LAION dataset, used to train Stable Diffusion [RBL^*^22]. A review of the search “data visualization” on this database returns a number of graphics with identifiable authorship, as well as graphics that violate common mores of visualization, e.g. 3D bar charts showing occlusion, distorted iconography, distracting gradients, and cluttered interfaces—all concerns listed in Tab. 1.

A variety of other tasks might be fulfilled at this stage. **P1**_*vis*_ described the potential of automatically reverse engineering data from an image. This work has been explored previously [PH17]. However, using a generative model might allow better extraction of data (not based on a predefined schema), but it may also introduce new, more difficult‐to‐address or identify forms of bias. Generative models could be used to construct useful situationally‐aware prompts (such as in the style of LitVis [WKD18]) that can help users reflect and consider how they are enshrining bias into their models of data. Finally, generative models are trained with large datasets (Fig. 6 left), and their results can greatly vary depending on this training. Curating input data can help reduce biases, personalize models, and increase the general quality of results. The complexity latent to this task type could be allayed by a generative model, which might be able to act as an assistant, such as Hynes et al.'s data linter [HST17], which could provide more dynamic or situational suggestions than heuristically‐motivated training‐data analysis tools.

### 4.2. Transforming

Once the data is established, it needs to be transformed in to a form that might be pliable for visualization. This process can involve wrangling, processing, introducing additional models (such as through regression or other advanced analytics), or countless additional approaches. Within each of these interleaved steps and stages, there are opportunities for a generative model to exert agency. For instance, prompts like “remove outliers” or “find missing data” presupposes particular structures of the data. While those might sometimes be unambiguous (for instance, a monthly calendar missing data from the weekends has clearly defined gaps), in other cases, it might be less clear (for instance, removing outliers might assume a particular model of the data distribution). Consider **cleaning data** (Fig. 7 center‐left); this stage might require removing duplicates or outliers, and converging on consistent naming—among a host of other sub‐activities. These repetitive processes prove to be something that can largely be automated [KW19], and so a generative model might usefully intervene by automatically creating transformations. Tools like Copilot [Git22] can already effectively assist in such a process when there is a human‐in‐the‐loop to help guide and curate the synthesis. Just as in the previous stage of our pipeline, yielding agency to a model runs the risk of introducing the model's biases into the generated content, which **P9**_*art*_ and **P17**_*vis*_ were particularly worried about. In addition, it may also risk out‐of‐domain schemas which, noted **P21**_*ML*_, may cause the suggestions to be irrelevant or incorrect. Further, the potential frictionlessness of this process may cause the user to overly trust the synthesized results, a huge issue for many participants (**P11**_*vis*_, **P12**_*vis*_, **P17**_*vis*_, **P19**_*vis*_), which may lead to difficulties in identifying errors down the line. In addition to acting as a tool to automated transform creation, generative models might also be used to evaluate whether or not a transformation was done well or if any steps were missing, in something akin to a spell checker for data analysis, which might be analogous to Wu et al.'s [WTC22] use of an LLM to analyze Vega‐Lite charts. While current LLMs do not consider data as part of their synthesis process, future models might do so to ensure that suggestions are appropriate to the data and task to be pursued (as in Fig. 2.

In addition, to the general task of cleaning data, generative models might be used at each incremental step of the cleaning pipeline, such as in creating regressions or identifying missing data. **P11**_*vis*_, for instance, was interested in augmenting financial datasets for research purposes: *“getting data of financial transactions can be very hard, which makes it hard to test our techniques”.* These pose similar benefits (like increasing the speed to accomplish a task or showing an unknown functionality) and risks (furthering user and model biases) as in the general case.

### 4.3. Visualizing

The most prominent stage in our pipeline is the visualization stage. Here, the modeled data is mapped to a visual encoding which will subsequently be presented to the user. As our work and participant pool is centered around visualization, participants observed the most risk in this domain but also the most opportunities. For instance, some participants (**P4**_*vis*_, **P6**_*vis*_, **P10**_*art*_, **P12**_*vis*_) highlighted the potential for harm in found graphics that do not handle sensitive content carefully (which is aligned to McNutt et al.'s [MHK21] descriptions therein). Most common models contain some form of safety system against pornography, gore, and deep fakes, but they are not perfect and can sometimes be circumvented by resourceful users [Tay23]. Yet, the same free‐form nature that can be potentially harmful also offers a deep well of potentially unfamiliar ideas and suggestions that might unset settled ideas.

One such example is the **beautification of visualization designs**, which while mostly aesthetic, could also be functional. From the selection of proper colors based on semantics [EAKM^*^22, HYC^*^22] to more extreme visual transformations, it was considered by participants to be mostly “*subjective*” (**P15**_*vis*_). However, it was recognized that as with Coelho's [CM20] “Infomages”, they do change the impact and interpretation of a visualization. For example, **P17**_*vis*_ commented on the value of beautification by considering the *“entertaining value of generative models for engaging users” (**P17**_vis_).* Works like BeauVis [HIDI22] could be used to orient the training of models for “aesthetic amplification”. Participants expressed enthusiasm towards this aspect but also doubts its feasibility. Most of the concerns related to lacking trust in the result of the model—indeed, **P8**_*vis*_ explained, there would be no way of knowing if the change in design would affect the data represented in the resulting visualization, or that the change would be appropriate assuming the process is done through a model that has no awareness of the data and the context of the user. In addition, they may also be useful for the construction of color maps that are adapted to the input data, such as for medical imaging or cartographic applications. These issues are further discussed in Sec. 5.1.

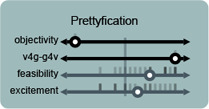

**Figure 8:**
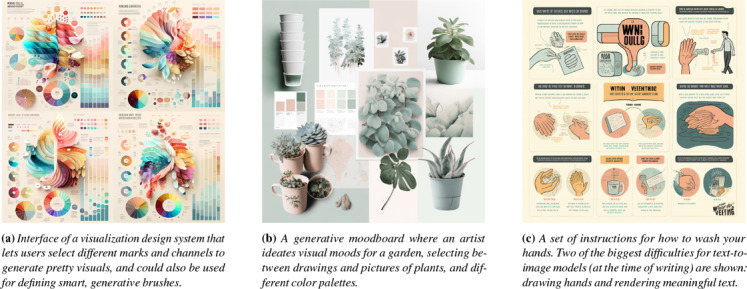
An illustration of possible manifestations of **Rapid Iterative Prototyping** with a generative model. We simulated a simple design sprint, starting with a vague idea of what we wanted, and collaboratively refined our prompts to obtain the above proofs of concept, each being a realistic visualization‐related use case. The model's strengths (aesthetics) and weaknesses (adhering to data) can be observed. While current technology is not able to elaborate on complex queries, these samples still usefully exemplify design thinking. They can serve as positive (“I like this”, “this works”, “I want this element”) or negative (“I don't like this”, “this is not what we want”, “we should remove this”) anchors.

Another example is **Moodboard creation**; an application where visualization supports the use of generative models. **P13**_*art*_ summarized this application as being like a *“Pinterest in the generative space”.* Many creative tasks begin with constructing mood boards, or a collage of images and text for inspiration and ideation. Generative models, aided by visualization and interactive techniques to navigate and query the latent space could become an infinite source of inspiration. Most participants believed developing such mood boards to be easy, because, besides Pinterest, there have been other examples of intelligent mood‐boards [KTBL^*^20]. **P21**_*ML*_ pointed out that pulling images from the latent space is still a very computationally‐intensive task, which limits this application. Fig. 8b shows what a generative mood board might look like for a particular design sprint.

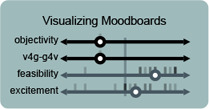

Generative models could be used to suggest types of charts that have fitting qualities to represent given data. Our discussion used an example of Tableau's **chart recommendation system.** which may have caused participants to think of this application as very feasible, as it already exists in some forms in commercial software. While participants were not very excited about generatively driven chart recommendation systems, however, some participants suggested adjacent notions. These included a variety of ideas, such as: *“Exploring the parameter space for design solutions to a task” (**P5**_vis_), “”Semantic“ chart recommendation” (**P5**_vis_), “Figuring out how to visualize multidimensional data that is hard to represent” (**P8**_vis_)*, and *“Search for novel visualization types” (**P8**_vis_).* Each of which seems like they have ample potential application throughout visualization.

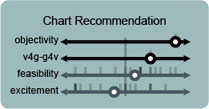

**Rapid iterative prototyping** is the process of rapidly changing parameters while designing or developing a prototype to test different ideas and quickly iterate over them. Most participants felt that it could be used both for supporting VIS and supporting the development of generative models, and both for exploring a design space (thus a subjective process) and more objective, pragmatic purposes. Participants were excited about being able to use generative models for rapid prototyping, and they believed it to be feasible. **P5**_*vis*_ suggested the IGMs could be used to “generate variations of a design”, rather than starting from scratch each time. A similar idea was expressed by **P19**_*vis*_ and **P10**_*art*_, in terms of *“exploring the parameter space for design solutions to a task”* and *“exploring potentialities of media”.* **P1**_*vis*_ and **P21**_*ML*_ made a connection between generative models and NLP‐based interfaces for information visualization. Similarly, **P14**_*vis*_ thought that it might be used as a *“search tool”*, which is similar to how users of Copilot perceive that tool [SGN^*^22]. **P12**_*vis*_ noted that *“prototyping entails different things and processes depending on the field”*, and was not confident that interaction or complex engineering solutions could be prototyped this way. We sketch this idea, and some of the highlighted opportunities here, in Fig. 8

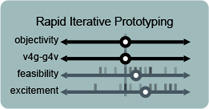

The concept of **chart embellishment** was presented to the participants as “generating parts of a visualization that don't really require data”—which participants (**P8**_*vis*_) also referred to as “chart junk”. We did not limit the concept to exclusively chart junk. For instance, the use of glyphs in visualization [SM14, YSD^*^22] or annotation, sometimes do not require data. Indeed, it is easy to see how IGMs could be used to generate iconic or symbolic encodings [Woo22], such as Chernoff faces [Che73], and support anthropo‐graphics [MJAD20]. At least two interviewees (**P12**_*vis*_ and **P17**_*vis*_) thought that this was the most probable “inevitable corporate application” to come soon.

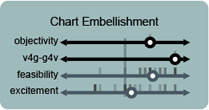

**Design styles being ripped off** was seen as inevitable, but opinions about how concerning it would be, were split. Some participants (**P1**_*vis*_, **P8**_*vis*_, **P15**_*vis*_) declared this wasn't a worry for them, and saw the issue as more of an advantage for them, while others cared deeply (**P4**_*vis*_, **P6**_*vis*_). The inevitability of it was often accompanied by a discussion on how this is already happening in the context of art and illustration. **P17**_*vis*_ commented that models are already doing this with illustrators and artists, and we should expect this for visualization too.

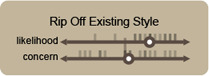

### 4.4. Interacting

The last pipeline stage comprises user interactions with data visualizations, or more generally, how they might *use* the created visualization. Such interactions are iterated and interleaved amongst other pipeline stages: seeing one chart may suggest constructing another, or may necessitate a return to the transformation stage to better clean the data.

An illustrative application of this potential is the Personalization (Fig. 7 right) of visual design to a user's tastes, tasks, and abilities. For instance, a model might act as an adaptor and automatically transform rendered visualization into more accessible versions that are tailored to a user's particular needs, such as color blindness or low visualization literacy (Fig. 8c). **P10**_*art*_ thought that being able to express your own *“personal aesthetics”*, such as by *“(training on your own data)”* would be useful. Graphics also might be personalized to how a user prefers to consume information. For instance, a manager might only like to see big number displays, while an analyst might like to see richer graphical forms. However, this also runs the risk that seeing only such display might accidentally cause hallucinations based on properties of the chart form [MKC20]. Such personalization also might be dynamic and make *“changes based on tracking user behavior and attention” (**P5**_vis_).* While such tools could help to obtain new perspectives, there is a risk that users could lose track of the overview of or sense of agency over [Hee19] the presented information.

Despite the value of these interactions, participants were concerned about both malicious and involuntary misleading information generated through the models. They cited the ability of these models to generate believable misinformation (**P1**_*vis*_, **P19**_*vis*_, **P21**_*ML*_) and mentioned deep fakes (**P7**_*vis*_, **P13**_*art*_) and fake news (**P10**_*art*_). **P17**_*vis*_ warned that this could also be involuntary, mentioning the possibility of *“irresponsible use of a black box”*, or just for the users of these models to be misled by their own results. **P14**_*vis*_ reiterated the dangers of using a black box without fully understanding it, saying that *“everything that is robust is obvious and everything that is obvious is robust”*, referencing Da's [Da19] argument that computational literary studies are merely counting exercises that only nominally interact with the text—a failure mode to consider in future systems design.

Finally, a variety of additional interactions and applications were suggested for these models. For instance, **P20**_*art*_ suggested that a *“content aware brush, painting enhancement”* interaction might be useful to get alternatives for a particular part of a chart. These models could be used to generate guidance, such as in the form of explanations. These might take the form of automatic summaries for charts (such as in the manner of Kanthara et al. [KLL^*^22]), or parts of charts, such as in a Tableau's Explain data feature [Tab22]—the advantage of the generative model is that results could be iteratively tuned and adjusted to taste. Such models may be able to synthesize animations or other interactions for previously static charts based on users demonstrating how they wish to interact with a chart or how they wish it to behave. Like other places in the pipeline where user preference can drive usage, this can lead to expressions of bias or what might be called an *AI‐filter bubble*, wherein the model reflects and amplifies the user's biases in a negative feedback loop. Further, high levels of intrusive automatic guidance could be distracting during the interpretation process. Similarly, if that guidance is delivered in a way that does not reflect the user's agency or previous decisions then it runs the risk of being perceived as impolite [Whi05].

## 5. Discussion

Despite some skepticism and critique clouding the introduction of generative models to new domains generally, participants in our interviews expressed tempered optimism and cautious excitement about the possibilities for applying these technologies to visualization. Participants were asked to think beyond current technical constraints, and even the most conservative visualization experts conceded the usefulness and inevitability of these technologies, for aesthetic, artistic, and disseminative purposes.

A common thread throughout our discussions was a sense of excitement: the huge technological leap to text‐to‐image models makes a compelling case for the potential of visualization in the age of generative models. We echo this sentiment and look forward to the dawning age of rich accessible illustrative visualization adapted to taste and to the task. Yet not every aspect of these models yields a delicious conclusion, which we discuss by considering cross‐cutting concerns (Sec. 5.1) and challenges (Sec. 5.2).

### 5.1. Concerns

**Figure 9:**
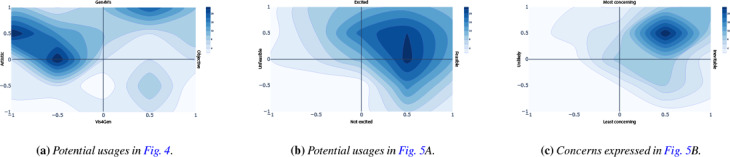
Aggregated participant responses. In (a) we see high interest in artistic applications of generativity to visualization creation. In (b) we see that the majority of concepts were considered feasible, and participants were mostly excited about them. In (c), similarly, the top‐right quadrant is dominant, indicating that the risks discussed were generally seen as concerning, and likely to happen.

Our participants' enthusiasm for these technologies is matched by their concerns. Participants mostly expressed that they were concerned about these technologies (Fig. 9c), but that they were, perhaps unfortunately, inevitable. Only **P18**_*vis*_, a visualization expert who is *“much more excited than worried about these technologies”*, considering most of the risks discussed *“not worth a thought”.* Participants typically had strongly‐held concerns, but there was not a general consensus of what was most worrying. Some viewed generative models as a form of Trojan horse, allowing the issues to creep in—such as amplification of bias, leaking of private data, deep fakes, and so on.

***Unreliable Results.*** The unreliability of results was one of the aspects of greatest concern. Most worries stemmed from the untraceability or unexplainability of the sources and suggestions. The stochastic nature of generative models and lack of semantic grounding makes it hard (or some might argue, impossible [MGSS21])toguarantee compliance to queries, which might produce undetectable or unverifiable wrong outputs. Although this is more serious for certain applications where precision is critical, it is also difficult to demonstrate that animage is original, which may incur possible copyright or ownership issues. To wit, **P6**_*vis*_ claimed that the risk of copyright violation would prevent them from generating even minor sections of a visualization. While some of these issues may have a technical solution (such as is promised in Amazon's yet‐to‐be‐released Code Whisperer [Ama22]), navigating the ethical elements beyond the legal ones remains a thorny task.

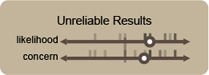

***Replacing Visualization Designers.*** Despite our own assumptions to the contrary, participants were generally unconcerned about the premise that generative models would replace designers of visualization. Similarly, participants were not worried about designs being stolen, which we found surprising given the comparable concerns in artistic [Vin22] and coding communities [Roo22]. **P6**_*vis*_ also observed that they do not believe they will be *replaced* per se, but for the expectations to change and for goalposts to be moved.

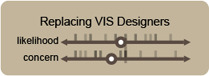

No participants questioned whether “can computers be creative?” Our participants may not view visualizations as artistic artifacts causing such issues to be viewed as irrelevant to our discussion. Alternatively, the lack of a consensus on what just defines creativity [FMBD18] may have made matters of AI creativity too murky to consider. Two practicing artists, **P13**_*art*_ and **P20**_*art*_, agreed that mastering generative tools will become an essential skill in their field, but espoused differing levels of optimism. While **P20**_*art*_ was enthusiastic about achieving an edge in his trade, **P13**_*art*_ is *“worried about the raising entry level for newcomers in an already cruel business”.* **P20**_*art*_ observed that tasks like illustrating land cards in *Magic: The Gathering* would likely be among the first jobs to be consumed by these technologies, as generative models can already create astonishing landscape paintings faster and more easily than experienced illustrators. **P9**_*art*_, **P10**_*art*_, and **P16**_*ML*_ (who are also involved in art, but are not practicing artists) offered curiosity and excitement about this new media, as well as ethical concerns—such as the use of publicly available art as training data without consent. The public perception of models that generate visualizations remains to be seen, but based on these initial reactions, we suggest that it may be less severe than the reception to generative art [Roo22]. Yet, the negatively perceived practices that led to the current generation of text‐to‐image models may have ‘poisoned the well’ for some groups. For instance, the visualization community on the vis.social Mastodon instance has specifically disallowed AI‐generated images that use text‐to‐image models such as MidJourney and Stable Diffusion, due to questions of provenance [Hen22].

### 5.2. Challenges

We next describe several high‐level challenges that we identified through out analysis.

***Data‐Constrained Generation.*** The inclusion of constraints is a prominent technical gap in diffusion models. For instance, most popular models (such as Midjourney [Mot22]) cannot currently deal with prompts involving quantities (such as “five dogs and two cats”), have trouble with text, and objects such as hands (Fig. 8c). Thus the substantially more constrained task of creating data visualizations is far off at the moment. Constraining the output of generative processes to data is not a new concept [Soc17, KM20], but it can be tricky to apply to the powered‐by‐noise diffusion process of which most text‐to‐image models are currently based. Yet, even once such techniques are possible, it might be long a time until such images can be reliably trusted—at least according to the views of our participants. Some skeptical participants emphasized this limitation, arguing that generative models have no place in objective visualization (Fig. 4, top‐right quadrant). This is aligned with how, despite the promise of recommendation engines to create elaborate images, analysts have primarily preferred to focus on simple charts with clear takeaways [BLF^*^22]. Still, we observed ample optimism in other interviewees, which suggests that when such a technology is manifest, it may be well received.

Beyond exploring how to better navigate and convey trust, there is ample room for improvement in generative models, such as from a performance perspective. For instance, the current resolution for generated images is still quite low, as higher resolutions require massive amounts of GPU memory. Simply by increasing the maximum resolution, we allow for higher frequencies to be rendered, which are essential for text and data visualization. We suggest that such issues indicate the value of investigating models that create a vector, rather than raster, images, as they may be less constrained by such technical limitations.

***Inheritance of AI Worries.*** By incorporating generative models into the visualization pipeline, we inherit many problems faced by the AI and ML communities. For instance, as we observed throughout Sec. 4, the issue of bias is a pressing concern. **P16**_*ML*_ observed that *“bias is a human problem, not a machine problem”*, so our approach to addressing these issues can not rely exclusively on technical solutions. Further, the current trend towards increasingly large models has meant that only companies with large resources are able to develop these models from scratch. Several participants (e.g. **P1**_*ML*_, **P12**_*vis*_, and **P14**_*vis*_) discussed the issue of centralization and democratization of these technologies. They observed that the current situation places a small number of firms in control of the data, allows them to exercise authority on what counts as bias, and may lead to censorship.

***Harmful Rationalization.*** The easier it is to create personalized, easily digestible, compelling, memorable, and beautiful visualizations, the harder it will be to guard ourselves against their influence. Misinformation becomes a bigger risk even without ill‐intent [MHK21], as a convincing visualization might lead a person to become overconfident over the knowledge gained from it [SEA22]. Their domineering form can make readers less questioning about conclusions [MCC20]. For instance, Galactica [TKC^*^22], a generative large language model meant to facilitate scientific inquiry, often gives incorrector biased answers to questions, but presented in an authoritative manner [Hea22].

Of course, when there exists actual ill‐intent in the use of these models the possible visualizations that can be generated from them become even more dangerous. Many participants (**P1**_*vis*_, **P7**_*vis*_, **P9**_*art*_, **P10**_*art*_, **P13**_*art*_, **P21**_*ML*_) worried about deep fakes and fake news, and that visualization powered by generative models has the potential to be a *“super fake news machine” (**P13**_art_).* Yet, one does not even need to use fake information, only to have an automated tool capable of choosing convenient half‐truths to support a position, querying the needed data and then creating beautiful and convincing visualizations that can be easily spread through unregulated social media channels. Further, most visual deception carried out on social media does not even require graphically tricking the user, rather data mirages like cherry‐picking are more than sufficient [LPLK23].

### 5.3. Limitations

As with any study, ours has limitations. We focused on the ideation process in our interviews to elicit ideas and opinions from our participants, which is an inherently speculative task. This conjectural nature likely influenced participants' responses, which would have been different if we had provided interactive demos. For instance, in the evaluation of concern versus likelihood, some participants reported not being concerned about something *precisely* because they thought it was unlikely.

Our study participants may not be representative of a wider or different population. As we focused on gathering the perspective from visualization researchers and practitioners, the other groups (artists, art historians, etc) were under‐represented in our sample. Some participants lacked familiarity with generative models and so had difficulty in discussing the default concepts. Similarly, our focus on domain experts may have biased our results. Future work should explore how these results extend to the public more generally.

The development of tools in this space continues to happen at a breakneck speed. During the course of our study, a number of notable models were released—such as MidJourney V4 [Mot22], Stable Diffusion 2.0 [RBL^*^22], Galactica [TKC^*^22], and ChatGPT [Ope23]. While the developments in these models are important, their behavior is not fundamentally different from the prior models, and so we believe that our findings will continue to apply as these models are further iterated upon. Our interviews only focused on IGMs, rather than the full scope of generative models. We chose to focus on image generation because of their cultural prominence at the time of our study, because of its functional similarity to the human act of producing data visualizations, and for its visually engaging artifacts, which we hoped would provoke more inspired participant responses.

### 5.4. Conclusion

In this work, we sought to chart the challenges and opportunities latent in the oncoming interweaving of data visualization and generative models, particularly those focused on text‐to‐image generation. We did so through a semi‐structured interview study with experts from a variety of domains and identified a number of areas of risk (such as the potential for harm that models trained on countless images hold) as well as areas of potentially ample reward (such as enabling easily accessible illustrative visualizations).

Shortly after we conducted our study, chatGPT was released to wide renown. This central achievement of this system is not a technical innovation (although the associated advances are non‐trivial), rather it is one of usability—leading to record‐breaking numbers of users engaging with an LLM for a wide variety of tasks [Reu23]. This popularity surfaced and extended discussions about some of the topics explored here, such as the consequences of generative AI systems for science more generally [SWVN23]. There is a new promising field of research in finding emergent abilities LLMs [WTB^*^23,KCK^*^23], which soon might be transferable to the visual domain [RKH^*^21], and using them as universal interfaces [SST^*^23].

Yet, we are never really prepared for the effects new technologies can have on ourselves, and on society. McLuhan observed that “*We look at the present through a rearview mirror. We march backward into the future*” [FM67]. However, we should not be afraid, because “*there is absolutely no inevitability as long as there is a willingness to contemplate what is happening*”. Through this work, we strove to contemplate what is happening, gathering the participants' experiences and opinions—their collective rearview mirrors—and then projecting them into the future. In doing so, we have tried to peer out from the walled garden of visualization [Woo22] and chart this land beyond the trees: its risks, challenges, and opportunities.

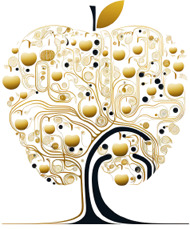

## Acknowledgments

We thank our anonymous reviewers for their helpful comments, as well as our study participants, without whom this work would not have been possible. This work has been partially supported by the European Commission under the project Humane‐AI‐Net (grant agreement 952026) and the Austrian Science Fund (FWF, grant P35767).

## Supporting information

Supporting Information
