# Supplementary material for: Doom or Deliciousness: Challenges and Opportunities for Visualization in the Age of Generative Models
Source: Comput Graph Forum. 2023 Jun 27;42(3):423–35. doi: 10.1111/cgf.14841 (PMC10946898; doi:10.1111/cgf.14841)
Supplement: Supplementary file 1 — Supporting Information [file CGF-42-423-s001.pdf]

Appendix A: Additional Details

In this appendix, we include additional information that we were unable to fit into the main body of the paper. These cover further details about our participants, the full structure of the interview, and the data we obtained through the interviews. Finally, in Fig. 11 we list the prompts used to create the imagery throughout the paper.

Participant backgrounds

As part of our onboarding, participants were asked to self-identify between Practice⟺Research and Artistic⟺Technical by shaping a circle over a plane, which we aggregate in Fig. 3. The resulting aggregation shows a variety of specialists and generalists over our covered spectra. This population includes 21 participants: 11 visualization researchers or practitioners (4 Ph.D. students, 4 postdocs, 3 researchers/designers, and 1 professor), the majority with over 5 years of experience and some with over 10 years, 3 artists each with over 8 years experience, 2 machine learning researchers, 2 HCI postdocs, and 2 art historians, one of which is also a philosopher and researches automated image-making. Fig. 3 shows a distribution of the aggregated self-identifications modeled as points. However, as can be observed in the miro interviews (end pages), each person had the freedom to shape a circle around this spectrum as desired, and participants varied widely in their choices of size and positioning.

Interview Format

The interviews were done over a miro board, and each interview was split into three phases: **onboarding** (introduction, self-identification), exploring **opportunities** (stages 1a, 1b), and understanding **risks** (stage 2). The two main forms of interaction were adding post-its with ideas to the board, and then ordering them relatively to the axis to answer questions, which are done while vocalizing thoughts and talking to the interviewer. Adding a post-it or splitting an existing one represents an act of ideation, and establishing relative positions are acts of comparative evaluation. The steps of the interview are organized so that subjects alternate between ideation and evaluation.

During the **onboarding**, the participants were presented with examples of experimental visualizations made with generative technologies by the authors, briefly discussed them, and then were asked to self-identify by shaping a circle over an axis (Fig. 10, top-left). This served to introduce the ideas that were to be discussed and to make participants accustomed to Miro and the interview format.

The **opportunities** and **risks** phases contain an initial population of concepts, which serves two purposes: to help the interviewee orient themselves in the proposed dimensions, allowing for easier ideation, and to force them to evaluate core ideas curated by the authors. Originally, the spaces were empty to avoid priming, but the pilot interviews showed us that eliciting novelty requires a lot of creativity and effort on the part of the interviewee, which could easily be blank. By having a set of relevant concepts in place we not only inspired subjects but also forced them to give us some input in the subsequent evaluation phase by ordering them in case they could not think of new ideas to add. The relevant stages for collecting insights were 1a, 1b, and 2, each with its own plane and conceptual goal.

Stage 1a. starts with participants being shown a populated space of concepts through the dimensions of purpose (vertical) vs. objectivity (horizontal). The purpose is defined between two polarities: generative models supporting visualization at the top, and visualization supporting generative models at the bottom. After an explanation and discussion of these ideas, participants are asked then to add their own, and the totality of ideas at the board will be transferred to the next stage (1b).

Stage 1b. sought to measure the relative excitement (vertical axis) vs the assumed feasibility (horizontal axis) of the ideas by spatially ordering them around. This was meant to gauge the participant's interest and belief that these applications could be useful and realistically integrated in the visualization process, or support it. Each quadrant was given a name based on its meaning to help the understanding of

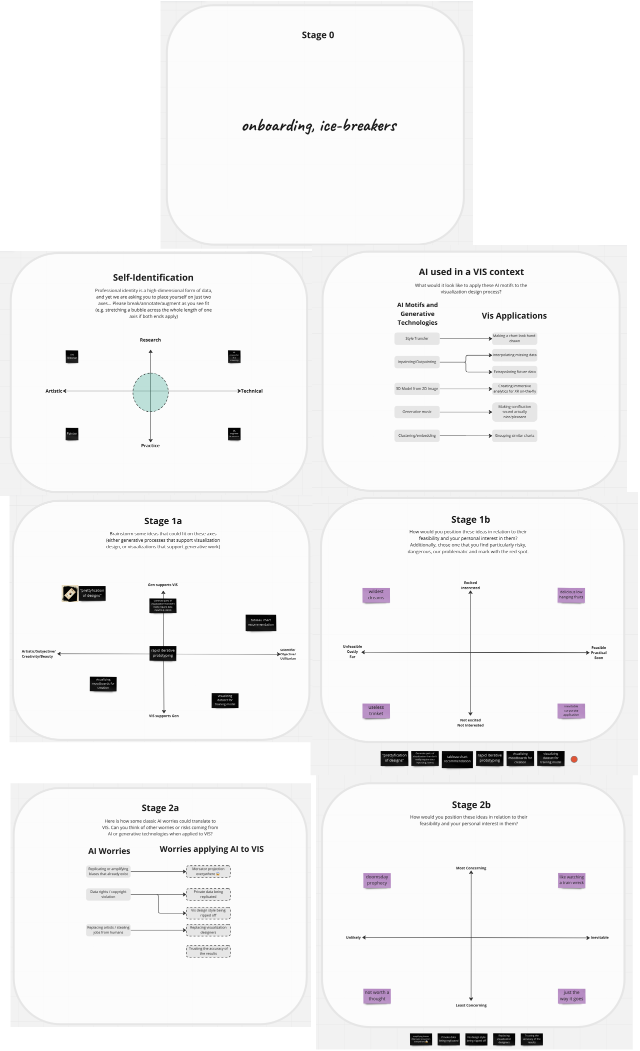

Figure 10: *A blank copy of the study instrument. Each participant was walked through each of the cells shown here, and add or sort sticky notes on the axes. The positioning of the sticky notes was then coded according to the axes, assigning each one a value between -1 and 1, yielding Fig. 9*

© 2023 The Authors.
Computer Graphics Forum published by Eurographics and John Wiley & Sons Ltd.

Figure 11: *Throughout the main paper we used a variety of IGM-created graphics, the prompts for which we list here.*

Figure	Tool	Prompt
1	Midjourney	Prompts appears in the figure, each use version 4
6a	Midjourney	*pastel mood board, data visualization, chart, infographic, interface –no monitor, globe, woman –v 4*
6b	Midjourney	*mood board data visualization, pastel colors, pictures of plants and mugs, pastel light tones –v 4*
6c	Midjourney	*pastel, data visualization, infographic, visual explanation of how to wash hands with steps, vertigo comics –v 4*
Inline Apple	Midjourney	*the forbidden fruit (apple) of generative models, enticing visualization researchers. minimalist design, Klimt, stencil, simplified, modernist, clean, white background*

the task. At the end of this stage, participants were also asked to indicate which one of the applications offered the biggest risk to them by overlaying a red dot on top of it. The top-right quadrant is especially important to us, indicating the most probable opportunities: ideas that participants are excited about and appear feasible, and this is why we named it **"delicious low-hanging fruits"**.

Stage 2. was intended to investigate the participants' fears and concerns about AI motifs applied to visualization, which would imply risk applications based on generative models. After being presented with a list of common "AI Worries", and some predefined risks for visualization, and then discussing them, the participant would be asked to add more ideas to the list. Then, once again, all ideas on the board would be moved to a plane, at this stage defined by concern (vertical axis) vs. likelihood (horizontal axis), where they had to be ordered comparatively. In this stage the quadrants are named relatively to the type of danger they represent: a concerning, but the unlikely scenario is a **"doomsday prophecy"**, but concerning and inevitable risks could be "like watching a train wreck", and tackling them should be treated as serious challenges.

The full artifacts of our interviews are included in the next pages.

© 2023 The Authors.

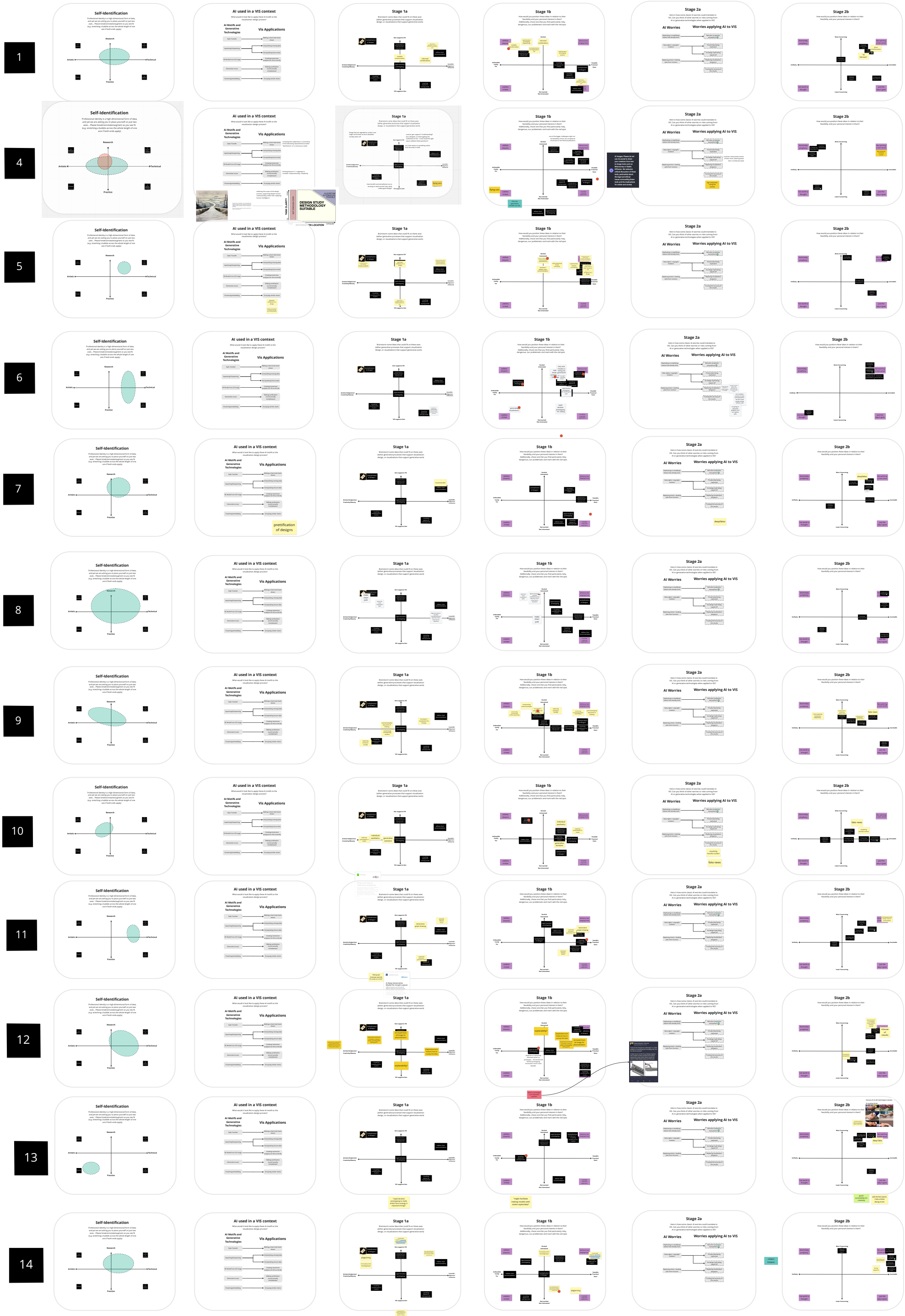

© 2023 The Authors.
Computer Graphics Forum published by Eurographics and John Wiley & Sons Ltd.

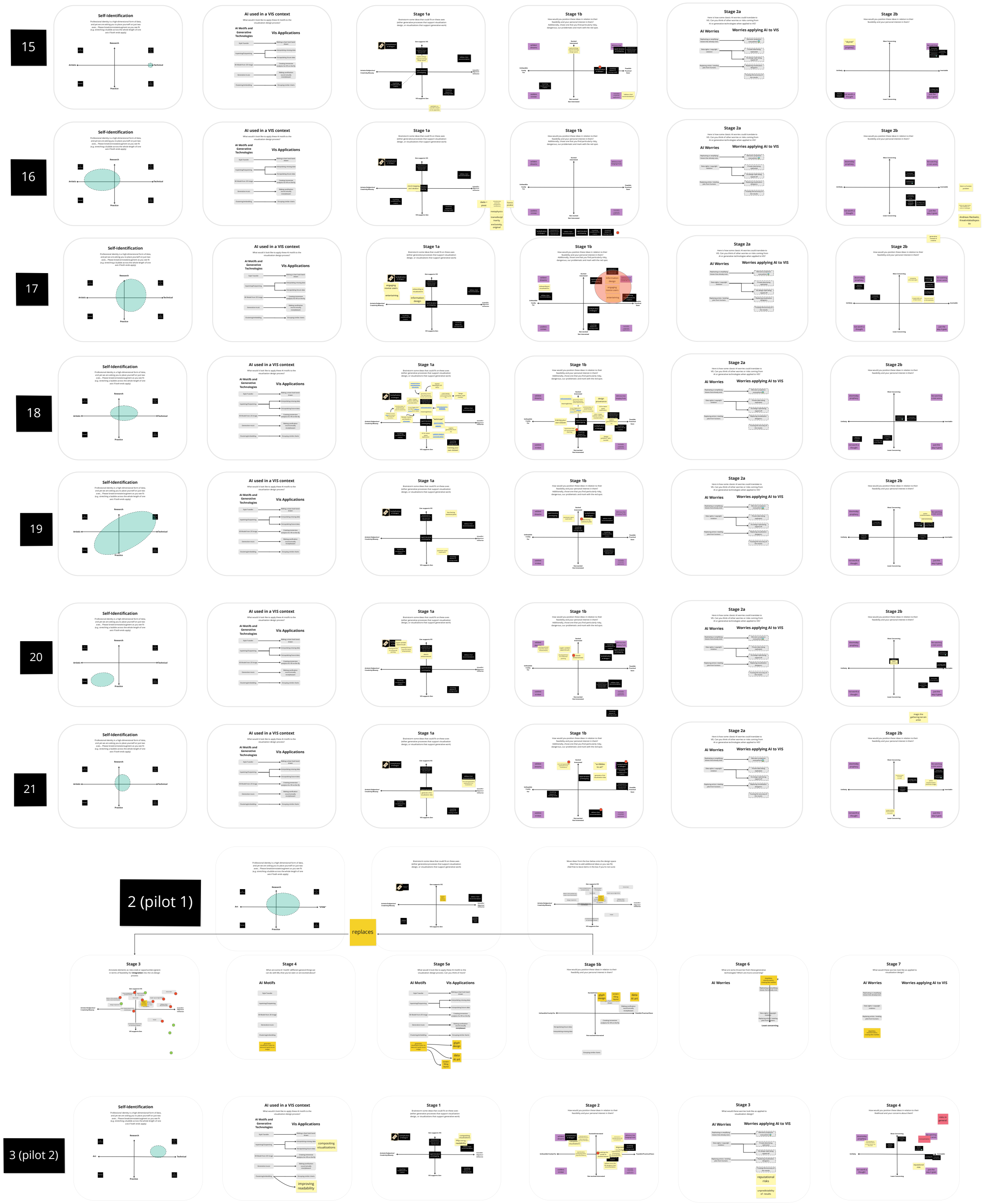

© 2023 The Authors.
Computer Graphics Forum published by Eurographics and John Wiley & Sons Ltd.
